# Application of ex vivo liver resection and autotransplantation in treating Budd-Chiari syndrome secondary to end-stage hepatic alveolar echinococcosis: A case series

**DOI:** 10.1097/MD.0000000000027075

**Published:** 2021-08-27

**Authors:** Cong Wang, Yiwen Qiu, WenTao Wang

**Affiliations:** a Department of Liver Surgery, Liver Transplantation Center, West China Hospital of Sichuan University, Chengdu, Sichuan Province; b Department of Hepatopancreatobiliary Surgery, Affiliated Hospital of Qinghai University, Xining, Qinghai Province, China.

**Keywords:** Budd-Chiari syndrome, ex vivo liver resection and autotransplantation, hepatic alveolar echinococcosis

## Abstract

**Background:**

Secondary Budd-Chiari syndrome (BCS) occurs due to a blockage in the liver caused by invasion or compression by a large lesion. Conventional treatments for BCS do not solve practical problems, wherease liver transplantation has been only applied as a last-resort therapy and as the only opportunity for a radical cure. We explored the feasibility of applying ex vivo liver resection and autotransplantation (ELRA) for the new indications of treating patients with end-stage hepatic alveolar echinococcosis (HAE). Our center has firstly proposed the idea and successfully treated the 49 patients with HAE. This article for the first time reports the application of ELRA in treating patients with BCS secondary to HAE.

**Methods:**

According to the degree of lesion invasion and surgical options, 11 patients were divided into 4 types. These 11 patients had large lesions that invaded the second and third hilum of the caudate lobe and involved the confluence of the hepatic vein and the inferior vena cava, suprahepatic vena cava, or at least 2 hepatic veins and led to secondary BCS. The aim of the present work was to report 11 patients with life-threatening diseases who underwent ELRA (ex vivo liver resection and autotransplantation) for secondary BCS, to propose a classification system for secondary BCS, and to suggest that secondary BCS is an indication for ELRA.

**Results:**

Eleven patients successfully underwent ELRA without intraoperative mortality. The median autograft weight was 690 g (440–950 g); operative time was 12.5 hours (9.4–16.5 hours); Postoperative hospital stay was 15 days (7–21 days). Clavien-Dindo grade IIIa or higher postoperative complications occurred in only 5 patients.

**Conclusions:**

This article for the first time reports the application of ELRA in treating patients with BCS secondary to HAE, not only provides new ideas for alternative treatments of secondary BCS, but also provides a classification system for secondary BCS. This article describes the technical process of outflow tract reconstruction and the experience for expanding the indications for ELRA. Our study demonstrated that ELRA is well feasible for treatment of BCS secondary to advanced HAE.

## 1 Introduction

Budd-Chiari syndrome (BCS) refers to the clinical syndrome caused by increased pressure in the hepatic vein (HV) and inferior vena cava (IVC) due to obstruction of hepatic venous outflow, which is caused by intrahepatic thrombosis or compression of lesions. Patients with BCS exhibit multiple manifestations including hepatomegaly, ascites, gastrointestinal hemorrhage, lower extremity edema and pigmentation, and even liver cirrhosis in the late stages of disease. Liver failure is the most severe complication that makes BCS sometimes fatal in some particular patients.^[1,2]^ The disease can be further classified into primary BCS caused by thrombosis and secondary BCS by lesion compression or blockage of hepatic outflow.

End-stage hepatic alveolar echinococcosis (HAE) is a rare cause of BCS. HAE is a lethal parasitic disease caused by infection with the larvae of Echinococcus multilocularis; this disease is characterized by tumor-like infiltrative growth leading to extensive intrahepatic destruction and even remote extrahepatic metastasis.^[3–5]^ The long asymptomatic onset of HAE often leads to a delayed diagnosis, and hepatic venous outflow occlusion caused by lesion compression may occur.

The conventional treatment for BCS is aimed at mitigating hepatic congestion and alleviating liver injury and its sequelae. Systematic anticoagulation and local thrombolytic therapy in select patients is the keystone of BCS treatment.^[2]^ Other therapeutic approaches including transjugular intrahepatic portosystemic shunt and surgical decompression of the portal system are also applicable for BCS.^[6]^ However, for BCS secondary to end-stage HAE, the continuous pathologies progress until outflow occlusion caused by the HAE lesion compression has not been resolved, and the conventional treatment modalities are invalid.^[7]^

Liver transplantation (LT) is considered a “rescue” treatment but is also a radical solution for BCS^[8–10]^ and is feasible in patients with secondary to end-stage HAE.^[11,12]^ Unfortunately, the utilization of LT is limited by the shortage of donors and potential posttransplantion relapse of HAE related to the mandatory use of immunosuppressants.^[13–19]^ In these circumstances, ex vivo liver resection and autotransplantation (ELRA) may provide an opportunity for radical treatment for BCS secondary to end-stage HAE as a possible alternative to LT. As one of the centers that pioneered this technique, we present our experience of treating BCS secondary to end-stage HAE, specifically with regard to the technical aspects and postoperative results.^[20–28]^ To the best of our knowledge, this is also the first study to consider secondary BCS as indication for ELRA.

## 2 Patients and methods

Ethics: The study was approved by the Ethics Committee of West China Hospital at Sichuan University (No. 2017–38) and was performed in accordance with the Declaration of Helsinki. Written informed consent was obtained from all patients.

### 2.1 Patients

From February 2014 to April 2018, a total of 49 patients with end-stage HAE underwent ELRA. Patients were qualified for ELRA if they had the distinguishing features: advanced HAE that was deemed “unresectable” with the use of traditional techniques because of difficulty exposing or removing the lesion and a lack of reconstruction techniques and materials; involvement of the hepatic hilar region, three HVs, and the retrohepatic vena cava or invasion of the tertiary branches of the portal veins and portal arteries requiring complex reconstruction with a prolonged ischemic time that the liver could not tolerate; good physiological state including normal liver and kidney function and extrahepatic echinococcosis lesions that could be surgically removed or controlled with albendazole. Eleven of the patients were preoperatively determined to have the complication of BCS. The patient information is presented in Table 1.

**Table 1:** Clinical data of the 11 patients.

Patient	Sex	Age, y	Pre-ELRA PTCD (n)	Lesion size, cm	PNM stage	Invade scope (hepatic vessels)	RLV, mL	RLV/SLV	Autograft mass, g	hepatomegaly	ascites
1	M	25	0	11.3	P4N1M0	Right and middle	600	1.07%	610	Y	N
2	F	41	0	19.2	P4N1M0	Right and middle	1000	1.38%	950	Y	N
3	F	41	1	14.7	P4N0M0	Right and middle the trunk of LHV	900	1.6%	880	Y	Y
4	M	32	1	11.7	P4N1M0	Right and middle the confluence region of LHV	600	1.13%	565	Y	Y
5	F	27	0	15	P4N1M0	Right and middle The confluence region of LHV	920	1.27%	900	Y	Y
6	M	44	0	20.4	P4N1M1	Right and middle	850	1.6%	840	Y	N
7	F	41	0	14	P4N1M1	Right and middle	440	1.06%	440	Y	N
8	F	41	0	22	P4N1M1	Right and middle the trunk of LHV	770	1.15%	750	Y	Y
9	F	36	0	14.5	P4N1M1	Right and middle The confluence region of LHV	700	1.03%	690	Y	Y
10	F	44	0	17.8	P4N1M0	Right and middle The confluence region of LHV	540	1.21%	600	Y	N
11	F	36	1	15.3	P4N1M0	Right and middle The confluence region of LHV	670	1.11%	630	Y	N

LHV = left hepatic vessel: PNM stage, the classifications P (parasitic in the liver), N (extension to neighboring organs), and M (distant metastasis) were recently developed by the European Echinococcosis Registry Network of the WHO Informal Working Group on Echinococcosis;^[39]^ estimated remnant liver volume, RLV = estimated standard liver volume, SLV, SLV = 706.2 × BSA+2.4, BSA = 0.007184 *×* Height (cm)0.725 *×* BW(kg)0.425.^[40]^

### 2.2 Preoperative evaluation

**Figure 1:**
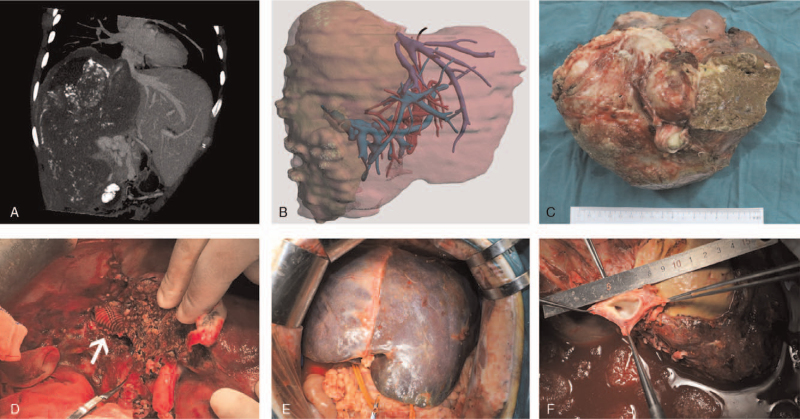
Preoperative assessment and surgical procedures for ex vivo liver resection and autotransplantation. (A) Preoperative CT scan shows a massive hepatic alveolar echinococcosis lesion occupying the entire right lobe and left medial lobe. (B) A 3D reconstruction of the patient's liver ^[29]^. (C) The lesion is approximately 20 cm × 15 cm; (D) An artificial graft was used to lengthen the outflow tract to prevent potential postoperative kinking. (E) Congestion of the residual liver. (F) The left hepatic vein has only 1 small hole left in it.

Further preoperative evaluations including computed tomography (CT) (Fig. 1A) and magnetic resonance imaging were performed to assess the anatomic characteristics of the lesion and the extent of hepatic venous outflow invasion. Patients with evidence of extrahepatic metastasis were also excluded. A 3-dimensional (3D) reconstruction system (IQQA-Liver; EDDA Technology, Inc, United States) was used for volumetric calculations and also facilitated preoperative surgical planning^[30–32]^ (Fig. 1B). All patients who underwent ELRA were also on the waiting list for DCD LT. After meticulous preoperative assessment and repetitive MDT discussions concerning the difficulties of conduit reconstruction and potential liver failure, the ELRA procedure was conducted with the preparation of a blood type-matched donation after cardiac death (DCD) liver graft. In addition, patient's family members were evaluated as donors for living donor liver transplantation (LDLT) when DCD LT was not available or if the waiting period was too long.Ultimately, no patients required salvage LT.

### 2.3 Surgical procedures

The technical details of ELRA have been described previously. Surgery was performed through a Mercedes incision. The liver was carefully explored and dissociated from adjacent adhesions. Once the liver was procured from corporeity and placed into an ice bath following ex vivo liver resection, the IVC was reconstructed with an artificial vascular graft (InterGard, InterVascular SAS, Inc, La Ciotat, France), and a Y-shape portocaval shunt was also established. Hemodynamic stability was temporarily maintained by these procedures. The inferior vena cava was resected “en bloc” with the liver and reconstructed using artificial blood vessels during the anhepatic phase, except for ascites, symptoms of portal hypertension were not obvious in these patients, because hepatic echinococcosis is a chronic parasitic disease that forms compensation during the course of the disease, and portal vein cavernous changes were observed in previous admitted patients. Patients with portal hypertension symptoms usually have liver insufficiency or abnormal coagulation function, which is not suitable for treatment of ex vivo liver resection and autotransplantation.

**Figure 2:**
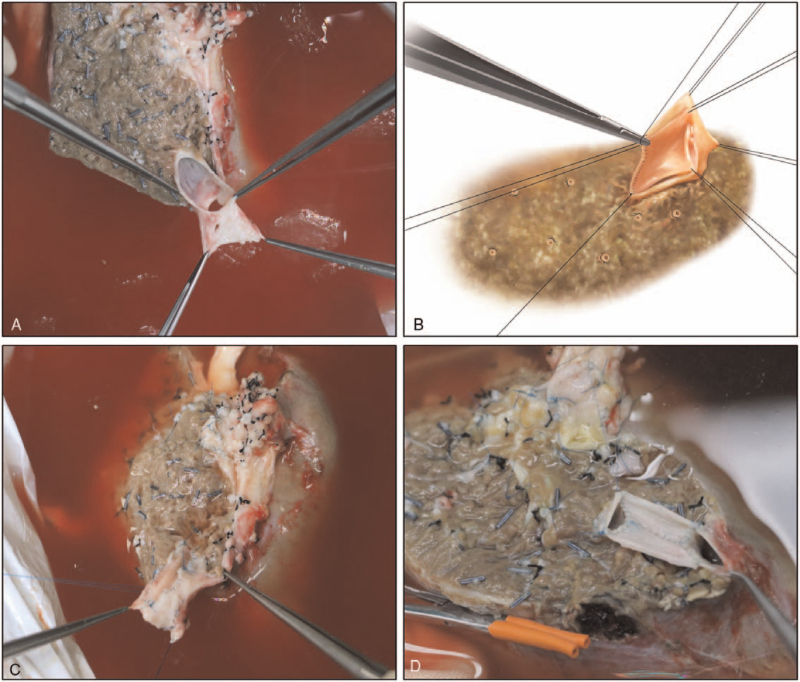
Reconstruction of the hepatic vein. Note: (A–C) The outflow tract of the residual liver required venoplasty, including repair of vessel defects with patches. (D) Extension of outflow stumps and unification of multiple stumps.

During the ex vivo resection, a Cavipulse Ultrasonic Surgical Aspirator (CUSA, Valleylab, Boulder, CO) device was used. The crucial conduit structures were carefully protected for subsequent reconstruction. After the lesion was totally removed (Fig. 1C), the outflow tract of the residual liver required venoplasty, including repair of vessel defects with patches (Fig. 2A–C), extension of the outflow stumps, and unification of multiple stumps (Fig. 2D) to facilitate further reconstruction procedures. In addition, when the outflow tract had a deficient length, an artificial graft was used to lengthen the outflow tract to prevent potential postoperative kinking (Fig. 1D). The objectives of outflow venoplasty are as follows: radical removal of the infiltrated portion and construction of a wider outflow tract to prevent postoperative hepatic venous outflow occlusion.

After performing ex vivo liver resection, the IVC was reconstructed before reimplantation of the residual liver. Reconstruction of the crucial conduits was performed in the following order: HV, portal vein, hepatic artery, and bile duct. Notably, an extended incision was made in the anterior wall of the vena cava to increase the diameter of the hepatic venous anastomosis.^[23]^

### 2.4 Postoperative management

**Figure 3:**
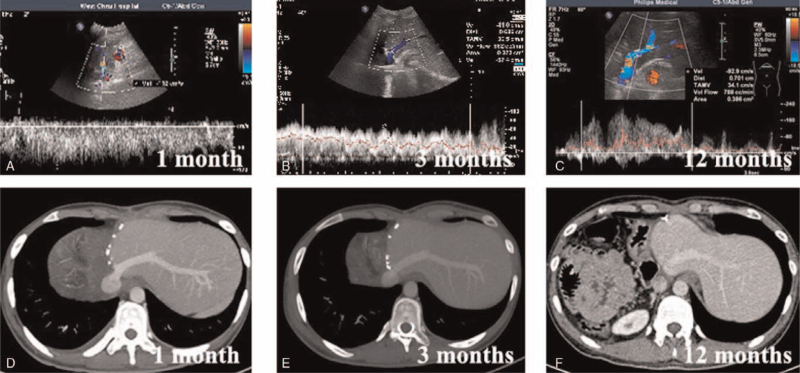
Results of postoperative CT scans and color Doppler ultrasonography.

Color Doppler ultrasonography was used to measure the diameter of the hepatic venous outflow tract for early detection of possible postoperative outflow obstruction (Fig. 3A–C). Once postoperative bleeding was excluded, a low-molecular-weight heparin sodium injection (0.4 mL q12 h) with the individual dosage adjusted according to the weight of the patient and the blood international normalized ratio (INR) was administered postoperatively until discharge. The patients were subsequently required to take warfarin sodium tablets orally (the individual dosage was adjusted according to the weight of the patient and the INR; the INR reference value was 2.0–3.0) for at least 3 months. All patients were administered albendazole (15 mg/kg/day) routinely for 2 years after ELRA.^[23,33]^ The patients returned for follow-up visits every 3 to 6 months after discharge (Fig. 3D–F).

## 3 Results

All the patients successfully underwent ELRA without intraoperative mortality. The median autograft weight was 690 g (440–950 g), the operative time was 12.5 hours (9.4–16.5 hours), and the postoperative hospital stay was 15 days (7–21 days). Postoperative complications of Clavien-Dindo grade IIIa or higher occurred in only 5 patients, including 1 patient who was discharged from hospital after multiple organ failure. Two patients developed outflow tract stenosis and improved after stenting. Biliary leakage occurred in 1 patient who improved after treatment with endoscopic nasobiliary drainage. Two patients had intestinal anastomotic bleeding, one of whom required a second operation, and the other improved after conservative treatment. The patient information is presented in Table 2.

**Table 2:** Intraoperative and postoperative surgical parameters of the 11 patients.

Patient	Combined resection	Hepatic vein reconstruction materials	Type	Operation time, h	Postoperative complication	Postoperative hospital stays, days
1	N	The trunk of LHV	I	11	N	12
2	N	The trunk of LHV	II	9.4	N	17
3	N	Autologous blood vessels Repairing with a patch	III	15	Infection of incision Intestinal anastomotic bleeding	19
4	N	The trunk of LHV	II	11	Outflow tract stenosis	15
5	N	The trunk of LHV	II	12	N	21
6	Part of the diaphragm	The trunk of LHV	II	12	N	18
7	N	The trunk of LHV/autologous blood vessels/repairing MHV	II	12.8	Biliary leakage	13
8	Spleen, right adrenal	Autologous blood vessels /repairing with a patch	III	16.5	N	7
9	A portion of right lower lung Right kidney	with plastic operation of 2 branches	IV	15	Kidney failure, liver failure	20
10	N	With plastic operation of two branches	IV	12.5	Intestinal anastomotic bleeding	15
11	Part of the diaphragm	the trunk of LHV	II	13	Outflow tract stenosis	10

LHV = left hepatic vein, MHV = middle hepatic vein.

**Figure 4:**
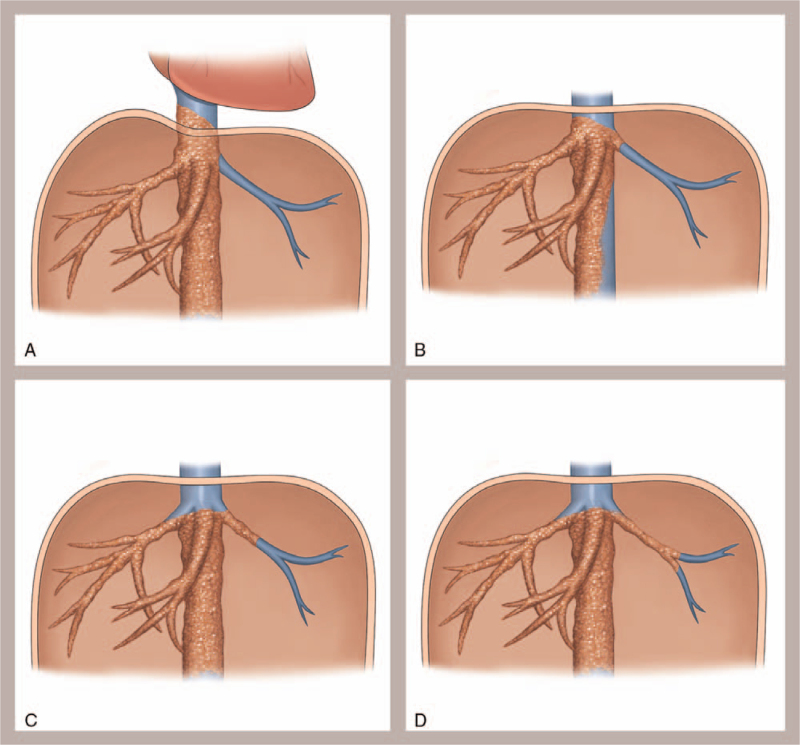
Patient classification by degree of disease. (A) Type I: Lesions invading the superior and inferior hepatic vena cava to the pericardium. (B) Type II: Lesions invading the confluence without affecting the main venous trunk of the residual liver. (C) Type III: Lesions invading the main hepatic vein trunk or hepatic interlobar branch; Type IV: Lesions invading the residual hepatic vein in the remnant liver segment.

According to the degree of lesion by invasion and operative technique, 11 patients were divided into 4 types to improve comprehension of the anatomical features of venous invasion and guide the reconstruction procedures (Fig. 4A–D). In patient 1, the lesion invaded the upper vena cava reached the right atrium and led to considerable difficulty during the anastomosis, and the diaphragm had to be opened during surgery. During the anhepatic phase, we choose an artificial blood vessel to anastomose with the right atrium to reconstruct the IVC. The anastomotic technique is very important and is the key to success. The diaphragm surrounding the vena cava was excised with electrocautery, and the pericardial space was widely exposed. The suprahepatic vena cava was also fibrotic and did not have enough length to allow an anastomosis; thus, a Satinsky clamp was placed diagonally on the right atrium without causing any arrhythmias, and the bottom of the atrium was cut 2 cm wide so that anastomosis could be performed. The fibrotic native vena cava was removed, and anastomosis between the right atrium and the artificial vascular graft was performed with 5/0 polypropylene sutures in a continuous fashion. The pericardium was closed at the end of the procedure, and the proximal aspect of the IVC near the diaphragm was curved 2 cm wide so that anastomosis could be performed. Patient 3, in whom the outflow tract of the residual liver was invaded, required additional plastic procedures to facilitate the reconstruction of the outflow tract because of the excessive removal of the HV during ex vivo radical resection. Depending on the length of the invasion, if necessary, a patch may be needed. All types of surgeries must be carried out with sufficient residual liver quality (Fig. 1E). For instance, in patient 7, the residual liver was small; the middle HV had to be repaired with a patch of autogenous vessels, and the left HVs were anastomosed with the IVC.

## 4 Discussion

The radical treatment of HAE is the prerequisite for treating BCS secondary to end-stage HAE. In addition to the infiltration of hepatic venous outflow, HAE is characterized by comprehensive invasion of multiple intrahepatic structures during the advanced to late stages of disease, making patients unsuitable for conventional surgical treatment.^[34]^ Our attempts at applying LDLT had acceptable outcomes; however, the inevitable limitations of LT including shortage of donors and requirement for immunosuppressive therapy, prompted us to utilize ELRA as an alternative to LT to treat patients with BCS secondary to end-stage HAE. The sophisticated strategies for hepatic venous outflow reconstruction further complicated the surgical planning and actualization of ELRA, which made it a challenge even for surgeons at experienced centers.^[35–37]^ This article is the first to report the application of ELRA for treating BCS secondary to HAE and provides new ideas for other treatments of secondary BCS and experience for expanding the indications of ELRA.

For end-stage HAE patients diagnosed with BCS, in addition to routine examinations, preoperative examinations focus on assessing the hepatic venous outflow to determine the extent of obstruction and infiltration and prepare for subsequent reconstruction procedures. The patency of the outflow tract requires careful inspection to identify evidence of possible compensatory outflow branches. In Patient 5, we carefully protected a small vein in the left ligamentum deltoideum that was considered as an important compensatory branch for hepatic venous outflow. After mobilizing the liver, the small compensatory outflow branch was ultimately ligated at the moment that liver was retrieved in vivo. This modality prevented the liver from excessive congestion and minimized the damage to the liver. Notably, severe disturbances in systemic hemodynamics were observed in almost all patients when we lifted the liver upwards during the mobilization; these disturbances led to temporary occlusion of hepatic outflow and a sharp decrease in the blood flow returned to heart (Fig. 1F). This phenomenon further emphasizes the importance of close cooperation between surgeons and anesthetists.

We have developed a classification system with various ELRA surgical proposals based on the extent of venous infiltration to improve comprehension of the anatomical features of venous invasion and guide reconstruction procedures. Type I included 1 patient (Patient 1) with lesions invading the superior and inferior hepatic vena cava extending to the pericardium(Fig. 4A); in these patients, the diaphragm must be opened during surgery, and even the right atrium may need to be included in the anastomosis. Type II includes patients with lesions invading the confluence without much influence on the main venous trunk of the residual liver (Fig. 4B). These patients can be treated with direct anastomosis of the residual HV trunk. Type III includes patients with lesions invading further into the main HV trunk or the hepatic interlobar branch (Fig. 4C). These patients need to be treated with residual HV branches and plastic surgery followed by anastomosis (Patient 3 and Patient 8). Type IV includes patients with lesions further invading the residual HV in the remnant liver segment (Fig. 4 D). These patients need to undergo repair with a patch for lengthening followed by anastomosis, (Patient 9 and Patient 10).

However, this classification scheme is not a rigid doctrine. In Patient 7, who was classified as Type II, a special modality was adopted, and 2 separate outflow tracts were reconstructed. According to preoperative simulations, a major vein that was originally a tributary of the middle HVs, was retained to ensure venous drainage of segment IV, in addition to the left hepatic venous outflow of the liver. This example reminds us that specific reconstruction plans should be individualized based on the results of preoperative examination combined with intraoperative findings. The reconstruction of the HV is an important topic in this procedure. The following 3 methods were used to reconstruct HV: total autologous vascular repair and reconstruction; allogeneic blood vessel cryopreservation; and artificial blood vessel repair. For patients with cryopreserved allografts, rejection may occur after surgery. The reconstruction technique of the HV using artificial blood vessels during surgery is relatively simple. However, postoperative abdominal infection can lead to vascular graft infection and the failure of the reconstruction of the HV; thus, the long-term patency still needs to be determined with clinical long-term follow-up observations.^[38]^

No complications above Clavien-Dindo grade III were associated with the outflow tract. Clavien-Dindo grade IIIa complications occurred in 2 patients who developed long-term outflow tract stenosis and improved after stenting. We speculate that this finding may be related to the length of the outflow tract. This result once again emphasizes the importance of ensuring the patency of the outflow tracts in ELRA. According to our experience, hepatic venous outflow reconstruction in ELRA should be performed in accordance with the following principles: a sufficiently wide orifice for outflow tract anastomosis must be ensure; the extended portion should not be too long to avoid kinking during postoperative liver hyperplasia; for patients with a long outflow tract, artificial grafts of a certain strength are rational for preventing outflow tract kinking during postoperative liver hyperplasia. Patient 4 had no reconstruction of the IVC because of the rich collateral circulation detected by preoperative venography. The hepatic outflow orifice was too far from the stump of the suprahepatic vena cava; thus, an artificial graft was modified and then used to complete the outflow tract reconstruction (Fig. 1 D).

This article for the first time reports the application of ELRA in treating patients with BCS secondary to HAE, not only provides new ideas for alternative treatments of secondary BCS, but also provides a classification system for secondary BCS. It describes the technical process of outflow tract reconstruction and the experience for expanding the indications for ELRA. Our study demonstrated that ELRA is feasible for treatment of BCS secondary to advanced HAE.

### 4.1 Uncited reference

^[29]^.

## Author contributions

Study conception and design, Interpretation of data, Critical revision: Wentao Wang

Drafting of manuscript: Cong Wang, Yiwen Qiu

**Supervision:** WenTao Wang.

**Writing – original draft:** Cong Wang.

**Writing – review & editing:** Yiwen Qiu, WenTao Wang.
